# Daily Optical Coherence Tomography Examinations after First Antivascular Endothelial Growth Factor Injections: An Interventional Case Series

**DOI:** 10.1155/2016/6971831

**Published:** 2016-05-05

**Authors:** Eduardo A. Novais, Emmerson Badaró, Flavio E. Hirai, Felipe Abdo Jorge, Paula Leal, Michel Eid Farah, Eduardo B. Rodrigues

**Affiliations:** Department of Ophthalmology, Federal University of São Paulo, 04023-062 São Paulo, SP, Brazil

## Abstract

*Purpose.* To evaluate daily spectral-domain optical coherence tomography (SD-OCT) changes in naive-treatment patients with diagnosis of exudative age-related macular degeneration (AMD) treated with intravitreous bevacizumab (1.25 mg), during a 30-day follow-up period.* Methods.* In prospective, interventional study, SD-OCT was performed daily for 30 days after the first intravitreal injection. The baseline, initial-decrease, minimal, and final central retinal thicknesses (CRTs) were assessed.* Results.* Nine eyes of nine patients with neovascular AMD were enrolled. The mean baseline CRT was 625.3 ± 182.5 *μ*m, and the mean final CRT was 383.4 ± 163.0 *μ*m (mean difference, 206.1 ± 167.6 *μ*m), a difference that reached significance (*P* = 0.006). After the first injection, the initial decrease in the CRT was seen as an average of one day after injection (mean CRT, 503.6 ± 189.10 *μ*m; *P* = 0.0431). The speed of the reduction in the CRT tended to decrease by day 17. The mean CRT was 336.5 ± 105.44 *μ*m and the mean minimal CRT on day 30 was 320.75 ± 96.38 *μ*m.* Conclusion.* The CRT decreased early after the first injection. We observed a tendency for reductions in the speed with which the CRT decreased by day 17 after the first injection, which may affect retreatment regime.

## 1. Introduction

Age-related macular degeneration (AMD) is the leading cause of irreversible blindness in patients older than 50 years in Western countries [1–3]. Vascular endothelium growth factor (VEGF) plays an important role in wet AMD [4]. As a consequence, many ophthalmologists worldwide have been using anti-VEGF drugs such as bevacizumab (Avastin, Genentech, Inc., South San Francisco, CA), ranibizumab (Lucentis, Genentech, Inc.), and aflibercept (Eylea, Regeneron Pharmaceuticals, Tarrytown, NY) [5] to attenuate the progression of this disease and in some cases restore vision [5–8].

Bevacizumab is a humanized IgG1 monoclonal antibody that binds and inhibits all isoforms of VEGF-A. The US Food & Drug Administration (FDA) approved the drug to treat metastatic colorectal cancer. Michels et al. proposed use of bevacizumab as an intravitreous treatment for exudative AMD in 2005, when they noticed that the visual acuity (VA) improved in patients treated with systemic bevacizumab for metastatic colorectal cancer [9]. Because bevacizumab is not FDA approved for intravitreous use, it is classified as off-label for this application. However, it is a low-cost alternative to ranibizumab or aflibercept.

The monthly intravitreal dose of bevacizumab was determined based on pharmacokinetics studies [10, 11]. However, to our knowledge, no clinical correlation has confirmed the best time for retreatment. Spectral-domain optical coherency tomography (SD-OCT) provides high-resolution anatomic images of retinal changes that can indicate the optimal timing of a new injection and facilitate a better understanding of the unknown pharmacokinetics of anti-VEGF drugs. To better understand the retinal changes, our research group performed daily SD-OCT examinations for 30 days in treatment-naive patients with active exudative AMD.

## 2. Methods

This was a prospective, interventional study conducted at the Department of Ophthalmology and approved by Federal University of Sao Paulo (UNIFESP), School of Medicine Institutional Review Board (IRB [#0345/10]). The research adhered to the tenets of the Declaration of Helsinki. Written informed consent was obtained before OCT examination in accordance with the UNIFESP IRB.

Nine patients were enrolled; the eligibility criteria were an age of 50 years or more, presence in a study eye of previously untreated active choroidal neovascularization (CNV) due to AMD, central macular thickness over 250 *μ*m, VA between 20/25 and 20/400 using the Early Treatment Diabetic Retinopathy Study (ETDRS) chart, and unremarkable past ocular history. All patients underwent a baseline complete ophthalmologic examination, SD-OCT using the Spectralis OCT (Heidelberg Engineering, Heidelberg, Germany), and fluorescein angiography (FA) to warrant a correct diagnosis.

After the baseline evaluation (day 0), bevacizumab (1.25 mg/0.05 mL) was injected into the vitreous cavity of each patient at the same visit. The patients were instructed to use topical antibiotics (moxifloxacin 0.3%) four times daily for 5 days. Commercially acquired bevacizumab was repackaged in glass vials in an aseptic filling facility.

Daily SD-OCT evaluations were performed for 30 consecutive days after the injection. After pupillary dilation, a 30-degree central macular line B-scan was performed with the automatic real-time follow-up function of eye tracking in the Spectralis OCT unit. Automated inner and outer retinal segmentation were used to assess central retinal thickness (CRT), and misidentifications of segmentation boundaries were confirmed and manually corrected to avoid segmentation errors that could have influenced macular thickness measurements. The baseline, initial decrease, and minimal and final CRTs were assessed. Patients who missed 10% of the examinations were excluded from the study.

### 2.1. Statistical Analysis

Data were collected daily from all patients after the first intravitreal injection and expressed as the mean (±standard deviation). The Wilcoxon signed-rank test was performed to compare the preinjection and postinjection retinal thicknesses. Stata version 11 (StataCorp, College Station, Texas) was used for all analyses. *P* values <0.05 were considered statistically significant.

## 3. Results

Nine eyes from nine patients (mean age of 74.4 years; 6 women, 3 men) with naïve-treatment active neovascular AMD were enrolled. All patients had active CNV confirmed by FA. Four patients were diagnosed as predominantly classic and five as occult according to the FA classification. The mean number of OCT examinations was 27.8 ± 0.78. No ocular or systemic side effects developed during the follow-up period. The mean baseline CRT was 625.3 ± 182.5 *μ*m; the mean final CRT was 383.4 ± 163.0 *μ*m (mean difference, 206.1 ± 167.6 *μ*m) between the two measurements (*P* = 0.006).  Table 1 shows the data from all patients.

**Figure 1:**
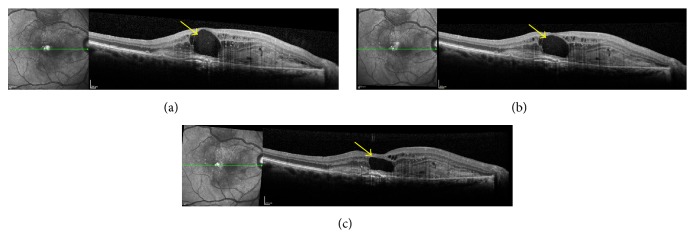
Cross-sectional one-line optical coherence tomography (OCT) B-scans of patient 8. (a) Pretreatment baseline visit. OCT B-scan shows a type 2 neovascular membrane presented as a hyperreflective image above the retinal pigment epithelium, associated with subretinal fluid and intraretinal cysts. (b) OCT B-scan follow-up with eye tracking after the first day after bevacizumab injection with decrease of subretinal fluid and intraretinal cysts. (c) Two-day follow-up shows an important decrease of the intraretinal cyst (yellow arrows).

**Figure 2:**
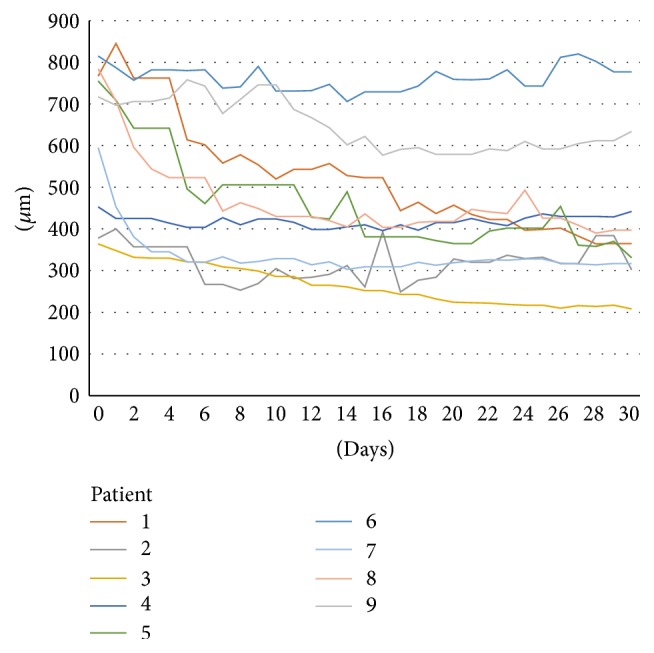
Daily central macular thickness measurements. The different colors represent each patient.

**Figure 3:**
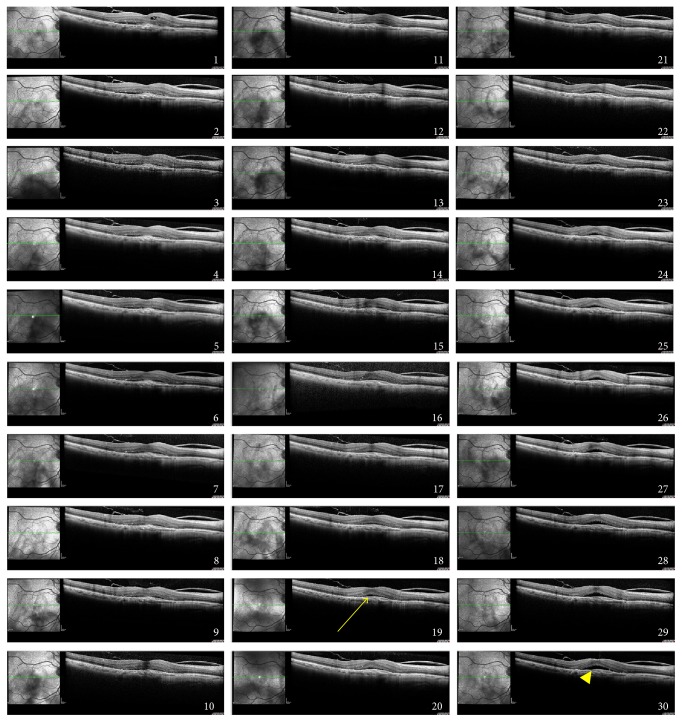
Daily cross-sectional one-line optical coherence tomography (OCT) B-scans over a 30-day period of patient 4. Immediately posttreatment decrease of central retinal thickness with rebound increase of subretinal fluid during follow-up, starting at day 18. On day 30, it is possible to identify the recurrent SRF (yellow arrowhead).

After the anti-VEGF injection, we noticed a decrease in the CRT (mean, 503.6 ± 189.10 *μ*m; *P* = 0.0431) starting on average on day 1 (Figure 1). On day 17, the mean CRT was 336.5 ± 105.44 *μ*m, and the mean minimal CRT on day 30 was 320.75 ± 96.38 *μ*m.  Figure 2 shows the daily progression of the CRT in each patient.  Figure 3 shows the 30-day OCT images from one patient.

On the predominantly classic classification, the mean CRT difference between the baseline and day 30 was 161.25 ± 171.10 *μ*m and for the occult classification was 242 ± 174.89 *μ*m (*P* = 0.4624). The mean CRT difference between the baseline and day 17 for the predominantly classic classification was 154 ± 119.16 *μ*m, and for the occult group this difference was 244.80 ± 140.68 *μ*m (*P* = 0.3272).

## 4. Discussion

Intravitreal injections of VEGF inhibitors have become the preferred treatment for a wide range of exudative eye diseases because of their antiangiogenic and antipermeability effects [12]. Since the initiation of intravitreous anti-VEGF therapy, ophthalmologists worldwide have created new hope for good treatment outcomes in patients with wet AMD. The improvements in VA and the decreases in subretinal fluid (SRF) and CRT were marked with this class of drug.

Although studies have confirmed that anti-VEGF drugs decrease the CRT and improve the VA in patients with AMD within a 4-week period, few pharmacokinetic studies have been conducted in humans to study intravitreous bevacizumab and ranibizumab [13–15]. Pharmacokinetic studies that have been performed have shown a rapid decrease in the circulating VEGF levels in the vitreous cavity, and even though the VEGF levels in humans remain undetected for 6 to 7 weeks after the anti-VEGF injection [13–15], the clinical changes are not in parallel with this result, since most increases in the CRT and SRF are observed during week 4. However, it is unknown if worsening of the clinical presentation occurs at that time or before, since in most practices the OCT evaluations of those patients have been performed only at the 4-week time point. The intravitreal half-lives of ranibizumab and bevacizumab in humans are estimated to be 7.9 and 9.73 days, respectively [16]. During the follow-up visits of patient 4, it was possible to identify subretinal fluid recurrence on the 18th day. Even though the final CRT from this patient was thinner than the baseline visit, there was a slow increase of the SRF from day 18 until day 30 (Figures 2 and 3). Also, the lack of incomplete response to anti-VEGF, in some patients (up to 10%) [17, 18], could be related to upregulation of proinflammatory factors, secondary to VEGF downregulation. In these incomplete responders, steroidal anti-inflammatory drugs (triamcinolone and dexamethasone) are well known for their positive effects on AMD [19–21].

The monthly injection was proposed based on a phase II study and animal models that addressed the safety and efficiency of that regimen [10, 11]. In a recent mathematical-model pharmacokinetic study, Stewart et al. showed that biweekly injections of anti-VEGF drugs presented higher trough levels and consecutive increases in the VEGF binding activity [22]. However, no clinical correlation has confirmed the optimal dosing. As a consequence, the precise interval of each injection is unknown.

More recently, there has been increasing concern about the development or enlargement of atrophic areas of the retina, glomerular disease, death, and arteriothrombotic events associated with repeated intraocular anti-VEGF [23, 24]. The 2-year results of the Comparison of AMD Treatment Trials (CATT) show that development of geographic atrophy (GA) was higher in the anti-VEGF monthly dosing group compared to the as-needed group [24]. On another study that analyzed the second-year results of the CATT, Sharma et al. showed that many features were associated with the largest decreases in VA at week 104, such as intraretinal fluid, extremely thin or thick CRT, an increase of CNV area, increasing foveal scar, and foveal GA. However, only intraretinal fluid was independently associated with worse VA at the second year of follow-up [25]. Grunwald and associates analyzed the growth of GA in the CATT and found no significant difference between subjects treated monthly and subjects treated as-needed [26]. In addition, since the CATT did not include a placebo treatment arm, we cannot determine whether anti-VEGF therapy has an effect on GA growth that is different from the natural history of GA developing in eyes with exudative AMD. Using gene-profiling assays to induce RPE-specific VEGF inactivation in mice, Kurihara et al. demonstrated that the decrease of VEGF affects the expression of multiple angiogenesis-related genes in isolated RPE-choroid preparations, leading to choriocapillaris loss and secondary cone photoreceptors dysfunction [27]. In another study, Saint-Geniez and colleagues showed that VEGF inhibition in mice leads to RPE dysfunction, accumulation of drusen-like basal deposits, and loss of barrier proprieties. Those findings are also often found in patients with AMD [28]. These changes preceded the development of GA [29]. However, there are no studies in humans showing a correlation between loss of retinal function and VEGF inhibitors. Thus, even though the risk of development or enlargement of GA may exist, it should be taken into consideration that undertreatment of wet AMD may have a higher impact on the VA.

**Figure 4:**
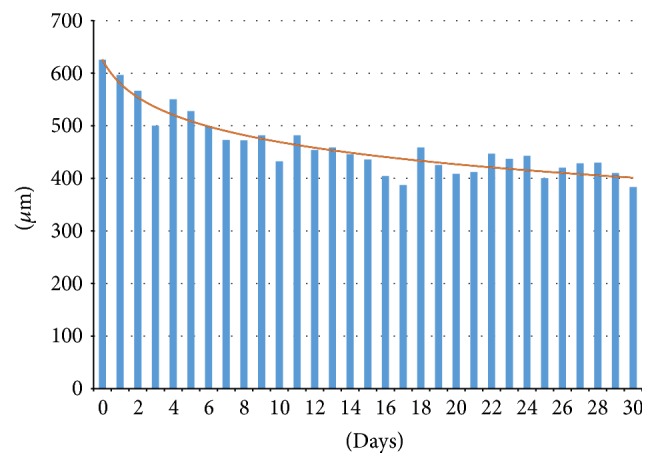
Mean CRT measurements (microns) during the 30-day follow-up.

In the current study, we identified an immediate initial decrease in the CRT and the amount of intraretinal fluid and SRF. The initial decreases in the CRT and SRF in the current study were consistent with previous pharmacokinetics studies and began the first day after the intravitreal injection of anti-VEGF medication. Hence, while the initial changes were similar to those reported previously, we observed a tendency for reductions in the speed with which the CRT decreased after day 17, which differed from the 6- to 7-week period of VEGF intravitreal inhibition in previous pharmacokinetics studies (Figure 4). However, even though our study described changes in CRT, it is important to highlight the fact that the functional improvements, such as an increase in VA, should be evaluated in combination with OCT for clinical evaluation.

In conclusion, since the current study showed different behavior of the intravitreal injections compared to studies that support current treatment regimen, we strongly support new studies of anti-VEGF intravitreal injections with a 2-week regimen compared to the standard 4-week regimen.

## Figures and Tables

**Table 1:** Patient data.

Patient	Gender	Age (years)	Baseline VA	Baseline CRT	First day of CRT reduction	Day of minimal CRT	Day of maximal CRT	Minimal CRT	Maximum CRT
1	M	82	20/400	767	2	30	2	355	845
2	W	80	20/100	378	2	17	1	249	400
3	M	74	20/80	364	1	30	1	208	364
4	M	69	20/60	453	1	16	1	361	453
5	W	83	20/200	755	1	30	1	331	755
6	W	65	20/400	815	1	15	1	729	787
7	W	66	20/150	595	1	14	1	303	453
8	W	72	20/200	784	1	29	1	397	707
9	W	79	20/400	717	1	16	1	577	697

M: man; W: woman; VA: visual acuity; CRT: central retinal thickness.

## Abbreviations

AMD: Age-related macular degeneration
VEGF: Vascular endothelium growth factor
FDA: Food & Drug Administration
VA: Visual acuity
SD-OCT: Spectral-domain optical coherency tomography
IRB: Institutional Review Board
CNV: Choroidal neovascularization
ETDRS: Early Treatment Diabetic Retinopathy Study
FA: Fluorescein angiography
CRT: Central retinal thickness
SRF: Subretinal fluid.
